# The HER2 phenotype of circulating tumor cells in HER2-positive early breast cancer: A translational research project of a prospective randomized phase III trial

**DOI:** 10.1371/journal.pone.0173593

**Published:** 2017-06-06

**Authors:** B. A. S. Jaeger, J. Neugebauer, U. Andergassen, C. Melcher, F. Schochter, D. Mouarrawy, G. Ziemendorff, M. Clemens, E. v. Abel, G. Heinrich, K. Schueller, A. Schneeweiss, P. Fasching, M. W. Beckmann, Ch. Scholz, T. W. P. Friedl, K. Friese, K. Pantel, T. Fehm, W. Janni, B. Rack

**Affiliations:** 1 Department of Gynecology and Obstetrics, Heinrich-Heine-University Hospital, Duesseldorf, Germany; 2 Department of Gynecology and Obstetrics, Ludwig-Maximilians-University Hospital, Munich, Germany; 3 Department of Gynecology and Obstetrics, University Hospital Ulm, Ulm, Germany; 4 Hospital Bremerhaven-Reinkenheide, Bremerhaven, Germany; 5 Hospital Ludwigsburg, Ludwigsburg, Germany; 6 Krankenanstalten Mutterhaus der Borromäerinnen, Trier, Germany; 7 Hospital Schwäbisch Gmuend, Mutlangen, Germany; 8 Praxis Dr. Heinrich, Fuerstenwalde, Germany; 9 Stat-up Statistische Beratung und Dienstleistung, Munich, Germany; 10 Department of Gynecology and Obstetrics in the National Center for Tumor Disease, University Hospital Heidelberg, Heidelberg, Germany; 11 Department of Gynecology and Obstetrics, University Hospital Erlangen, Friedrich-Alexander University Erlangen-Nuremberg, Comprehensive Cancer Center Erlangen-EMN, Erlangen, Germany; 12 Hospital Bad Trissl, Bad Trissl, Germany; 13 Institute for Tumor Biology, Center of Experimental Medicine, University Medical Center Hamburg-Eppendorf, Hamburg, Germany; Universita Campus Bio-Medico di Roma, ITALY

## Abstract

**Background:**

HER2 is one of the predominant therapeutic targets in breast cancer. The metastatic selection process may lead to discrepancies between the HER2 status of the primary tumor and circulating tumor cells (CTCs). This study analyzed the HER2 status of CTCs in patients with HER2-positive primary breast cancer at the time of diagnosis. Aim of the study was to assess potential discordance of HER2 status between primary tumor and CTCs, as this may have important implications for the use of HER2-targeted therapy.

**Methods:**

The number and HER2 status of CTCs out of 30ml peripheral blood were assessed in 642 patients using the CellSearch System (Janssen Diagnostics, USA). The cutoff for CTC positivity was the presence of at least 1 CTC, and the cutoff for HER2 positivity of CTCs was the presence of at least 1 CTC with a strong HER2 staining.

**Results:**

258 (40.2%) of the 642 patients were positive for CTCs (median 2; range 1–1,689). 149 (57.8%) of these 258 patients had at least 1 CTC with strong HER2 staining. The presence of HER2-positive CTCs was not associated with tumor size (*p* = 0.335), histopathological grading (*p* = 0.976), hormone receptor status (ER: *p* = 0.626, PR: *p* = 0.263) or axillary lymph node involvement (*p* = 0.430). Overall, 83 (32.2%) of the CTC-positive patients exclusively had CTCs with strong HER2 staining, whereas 31 (12.0%) had only CTCs with negative HER2 staining. Within-sample variation in the HER2 status of CTCs was found in 86 (57.8%) of the 149 patients with more than 1 CTC.

**Conclusion:**

This study demonstrated that discordance between the HER2 expression of CTCs and that of the primary tumor frequently occurs in early breast cancer. Future follow-up evaluation will assess whether this discrepancy may contribute to trastuzumab resistance.

## Introduction

The expression of HER2 is a poor prognostic factor and is associated with reduced overall survival (OS) in patients with breast cancer (BC)[1]. After highly effective HER2-targeted therapy became available, the outcome of patients with HER2-positive BC significantly improved[2]. However, up to 70% of patients who receive adjuvant HER2-targeted therapy (trastuzumab) after chemotherapy experience disease progression due to both de novo and acquired resistance[3]. Intratumoral heterogeneity in HER2 gene amplification may occur in 16%-54% of patients and may explain unexpected failures of adjuvant HER2-targeted therapy[4–6]. These failures are often associated with an equivocal (2+) HER2 status as assessed using immunohistochemical staining (IHC)[7]. Nevertheless, even tumors with clear HER2 overexpression (as evidenced by 3+ IHC results or by gene amplification using FISH) may fail to respond to HER2-targeted therapy[8,9]. Furthermore, the HER2 status of patients may change during disease progression[10,11], which may alter tumor responses to HER2-targeted therapy.

Reevaluating HER2 expression during disease progression is essential for adjusting the therapy to the current tumor status. Biopsy has been established for the assessment of HER2 expression in metastatic BC (MBC). Because of the lack of solid tumor tissue (primary tumor or metastasis) during follow-up in early BC patients, no sufficient marker is currently available. Determination of the HER2 status can be performed using circulating tumor cells (CTCs) as a “real-time biopsy” in the peripheral blood of BC patients[12–14]. Additionally in studies that used the CellSearch^®^ System, CTCs were found to be highly predictive of the OS and progression-free survival in MBC[15–17] and early BC[18].

In this study, we compared the HER2 expression of CTCs with that of the primary tumor in patients with HER2-positive early BC, which was determined after surgical intervention but before the start of systemic therapy. Differences in HER2 expression may explain unexpected failures of HER2-targeted therapy and reinforce the need to reevaluate HER2 expression during the course of early disease to determine the appropriate use of HER2-targeted therapy.

## Patients, materials and methods

### Patient population

This study was performed as a predefined translational research project of the SUCCESS B trial. From 2008–2011, a total number of 793 patients were enrolled in the SUCCESS B trial. The trial included 129 participating centers in Germany. The SUCCESS B trial was an open-label, multicenter, randomized, controlled, phase III study that compared the disease free survival (DFS) of patients who were treated with 3 cycles of epirubicin-fluorouracil-cyclophosphamide (FEC) chemotherapy, followed by 3 cycles of docetaxel (D) chemotherapy every three weeks (q3w), versus 3 cycles of FEC, followed by 3 cycles of gemcitabine-docetaxel (GD) chemotherapy q3w. At the end of chemotherapy, all of the patients had received adequate anti-HER2 treatment according to the study protocol. Eligible patients were confirmed to have HER2-positive invasive early BC with histopathological proof of axillary lymph node metastases (pN1-3) or high-risk node-negative disease, which was defined as a pT ≥ 1c, a histopathological grade ≥ 2, an age ≤ 35 years or a negative hormone receptor status. Categorization of the tumor stage at primary diagnosis was performed according to the tumor node metastasis (TNM) classification system that was revised by the American Joint Committee on Cancer (AJCC)[19].

Determination of the phenotype of the primary tumor was performed at each local laboratory of pathology according to the ASCO/CAP criteria for hormone receptor and HER2 determination using IHC (3+) or fluorescence *in situ* hybridization (FISH). The phenotypic results were obtained from the laboratory reports. All of the patient data were collected from a central database and exported after several data cleaning steps were performed at the beginning of 2013.

Patients were enrolled in the study no later than 6 weeks after the complete resection of the primary tumor. Written informed consent to participate in the SUCCESS B trial was obtained from all of the patients. The trial was approved by the ethical committee of Ludwig-Maximilians-University, Munich, Germany (EudraCT Number 2007-001094-29) and listed at ClinicalTrials.gov (NCT00670878). This trial was conducted according to the most recent version of the Declaration of Helsinki and the International Conference on Harmonization Good Clinical Practice Guideline, 1998.

### CTC detection

As part of the translational research project of this trial, we drew peripheral blood from the trial participants that provided written informed consent for the adjunct translational research program (not mandatory for recruitment in the SUCCESS B study) before the start of adjuvant chemotherapy. A total of 30 ml of blood was taken for the detection of CTCs. The semi-automated CellSearch^®^ System (Janssen Diagnostics, LLC, USA), which consists of the CellTracks® AutoPrep® System and the CellTracks® Analyzer II®, was used for the isolation and enumeration of CTCs. This CTC detection method is based on the use of antibody-coated magnetic beads for the immunomagnetic isolation of tumor cells that express the epithelial-cell adhesion molecule (EpCAM). This method has been previously published[15]. In brief, the blood samples were drawn into CellSave tubes (3 x 10 ml) that contained a cell preservative and EDTA. After shipment at room temperature, the samples were analyzed within 96 hours of collection at the central cancer immunological laboratory at the University of Munich. The blood of three CellSave tubes was pooled (for more information about the pooling step please refer to the detailed description provided as S1 File). After pooling, the blood was centrifuged for 10 minutes at 800 x *g* and a dilution buffer was added after the plasma was removed. In a syringe with a valve and tubing, this mixture was overlaid on 6 ml of Histopaque (Sigma, Germany) and centrifuged for 10 minutes at 400 x *g*. Subsequently, the CellTracks AutoPrep System and the CellSearch Epithelial Cell Kit (Janssen Diagnostics, USA) were used to process 7.5 ml of sample that contained the buffy coat and mononuclear cells. The CTCs were labelled with the fluorescent nucleic acid dye 4,2-diamidino-2-phenylindole dihydrochloride to stain the nuclei. Fluorescently labelled monoclonal antibodies that were specific for epithelial cells (cytokeratin 8,18,19-phycoerythrin) and leukocytes (CD45-allophycocyanin) were used to distinguish between these cell types. CTC identification was performed by two independent trained individuals using the CellTracks Analyzer II, a semiautomated fluorescence-based microscopy system. Nucleated cells that lacked CD45 and expressed cytokeratin were defined as tumor cells. Additional morphological criteria, i.e., a round or oval shape and a size ≥ 5 μm, were considered. A recent analysis demonstrated the good accuracy, precision, linearity, and reproducibility of the system[20].

**Fig 1:**
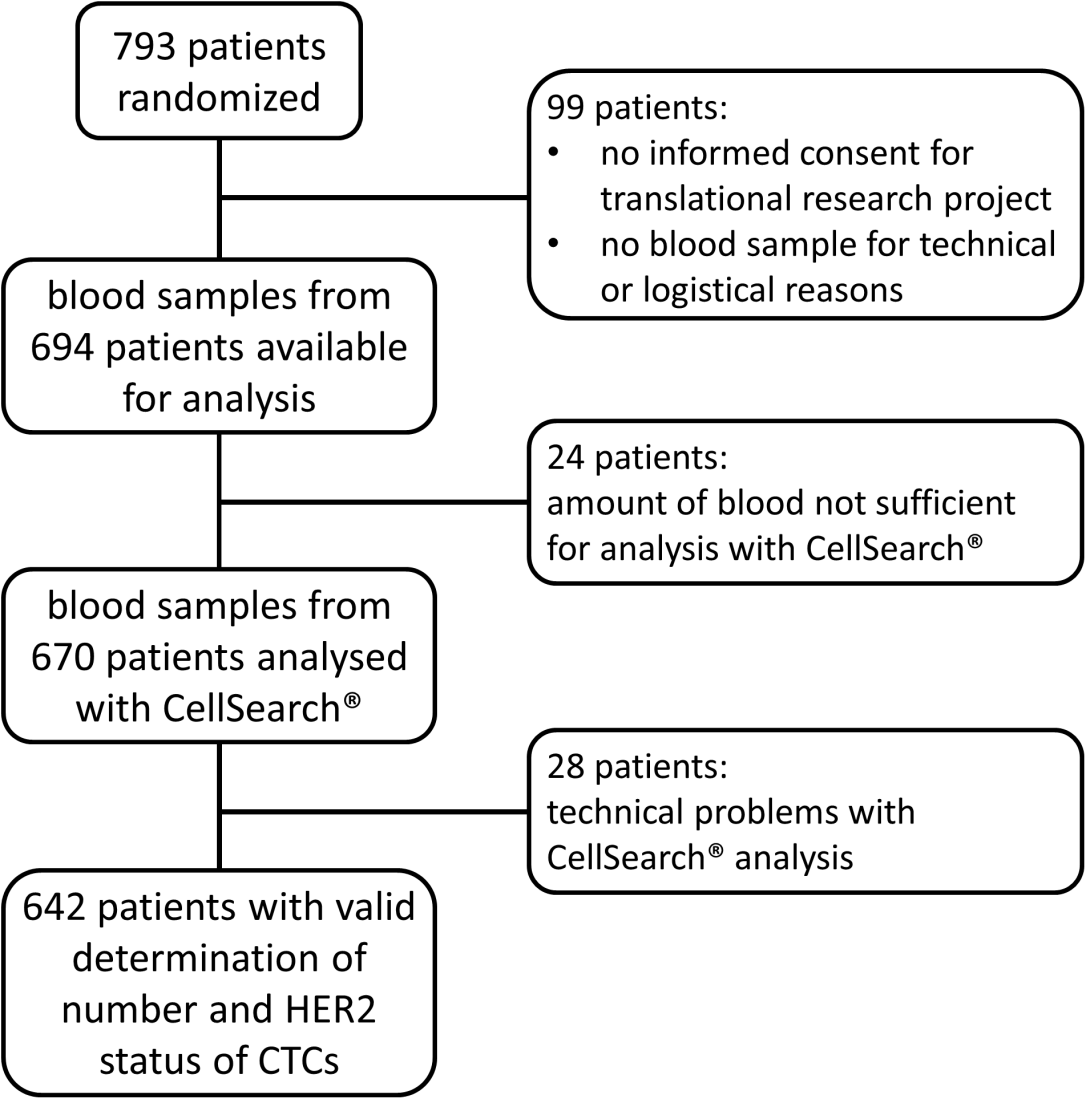
Flow chart illustrating study flow and patient selection.

Out of 694 blood samples available for analysis, 28 samples could not be analyzed due to technical problems with the CellSearch^®^ System, and 24 probes could not be evaluated because of a lack of sufficient amounts of blood (7.5 ml was the minimum amount required as established for MBC). Thus, we obtained valid measurements for the number and HER2 status of CTCs from 642 patient samples. Fig 1 illustrates the patient selection process for this study.

### Determination of the HER2 status of CTCs

FITC-labeled anti-HER2 antibodies were used to further characterize the HER2 expression of CTCs using the CellSearch System according to the manufacturer’s instructions (CellSearch tumor phenotyping reagent HER2; Janssen Diagnostics). The HER2 status of the CTCs was determined according to specific criteria by Riethdorf et al. To establish a standard for HER2 immunostaining, Riethdorf et al. spiked blood samples from healthy donors with approximately 500 breast cancer cells from cell lines with a known HER2 status (MCF-7, BT20, T47D, SK-BR-3, BT474 and MDA-MB-453) and processed these cells using the CellSearch System. In parallel, these cell lines were analyzed using immunocytochemistry and FISH to detect HER2 expression. HER2 gene amplification was determined by FISH, which counted the HER2 and centromere 17 signals. The ratio between the mean numbers of HER2 and centromere 17 signals determined the HER2 gene amplification. Riethdorf et al. distinguished between negative (-), weak (+), moderate (++) and strong (+++) HER2 staining according to the matched cell lines that were known to express HER2 and were correlated with the FISH analysis[21].

Using these criteria, only CTCs with a strong (+++) HER2 signal were defined as HER2-positive in this study. The cutoff for CTC positivity was the presence of at least 1 CTC per 30 ml of blood, and the cutoff for HER2 positivity in CTCs was the presence of at least 1 CTC with a strong (+++) HER2 signal.

### Statistical analysis

Descriptive statistics for all of the categorical data are summarized in frequency tables that present the absolute and relative frequencies, whereas the continuous but not normally distributed variables are described using median and range. Associations between the patient and tumor characteristics and the presence of CTCs and HER2-positive CTCs were analyzed using the chi-squared test. To assess which patient and/or tumor characteristics predict the presence of HER2-positive CTCs, we used a multivariate logistic regression model with presence of HER2-positive CTCs (yes/no) as binary response variable. The Cochran-Armitage test for trend was used to test for an association between the number of CTCs in a sample and the presence of HER2-positive CTCs. The correlation between the number of CTCs that were detected in a sample and the percentage of HER2-positive CTCs in that sample was analyzed using Spearman’s rank correlation coefficient. Finally, the binomial test was used to compare the proportions of HER2-positive and HER2-negative CTCs in patients with one CTC only. All of the reported *p*-values were two-tailed, and all of the calculations were performed using IBM SPSS Statistics (IBM Corp. Released 2010. IBM SPSS Statistics for Windows, Version 19.0. Armonk, NY).

## Results

### Prevalence of CTCs in patients with HER2-positive early BC

Valid measurements of the presence and HER2 status of CTCs before adjuvant chemotherapy were available for 642 patients. Of these patients, 258 (40.2%) were positive for CTCs (median = 2; range 1–1,689): 109 patients (42.2%) had one CTC, 65 patients (25.2%) had two CTCs, 38 patients (14.7%) had three CTCs, 12 patients (4.7%) had four CTCs, and 34 patients (13.2%) had five or more CTCs. Approximately half of the patient population had a pT1 tumor. Overall, 43% of the patients had lymph node involvement and the majority of patients had high-grade (G3) tumors. Of all the patients, 59% and 53% had a positive estrogen receptor (ER) or positive progesterone receptor (PR) status, respectively. No significant association was observed between the presence of CTCs and any of the clinicopathological parameters that were investigated (chi-squared test, all *p* > 0.05; see Table 1).

**Table 1:** Associations between presence of CTCs, patient characteristics and clinicopathological characteristics of the primary tumor.

Clinical variable at baseline	Total N = 642 (%)	CTC-negative N = 384	CTC-positive N = 258	*P*-value
**pT**				**0.480**
1	310 (48.3%)	190 (49.5%)	120 (46.5%)	
2	273 (42.5%)	154 (40.1%)	119 (46.1%)	
3	25 (3.9%)	16 (4.2%)	9 (3.5%)	
4	9 (1.4%)	4 (1.0%)	5 (1.9%)	
missing	25 (3.9%)	20 (5.2%)	5 (1.9%)	
**pN**				**0.510**
0	339 (52.8%)	204 (53.1%)	135 (52.3%)	
+ (1,2,3)	278 (43.3%)	160 (41.7%)	118 (45.7%)	
missing	25 (3.9%)	20 (5.2%)	5 (1.9%)	
**Histology**				**0.235**
Ductal	232 (36.1%)	128 (33.3%)	104 (40.3%)	
lobular	23 (3.6%)	12 (3.1%)	11 (4.3%)	
Other	360 (56.1%)	222 (57.8%)	138 (53.5%)	
missing	27 (4.2%)	22 (5.7%)	5 (1.9%)	
**Grading**				**0.780**
1	7 (1.1%)	5 (1.3%)	2 (0.8%)	
2	230 (35.8%)	136 (35.4%)	94 (36.4%)	
3	375 (58.4%)	219 (57.0%)	156 (60.5%)	
missing	30 (4.7%)	24 (6.3%)	6 (2.3%)	
**ER status**				**0.924**
negative	237 (36.9%)	140 (36.4%)	97 (37.6%)	
positive	380 (59.2%)	223 (58.1%)	157 (60.9%)	
missing	25 (3.9%)	21 (5.5%)	4 (1.6%)	
**PR status**				**0.986**
negative	272 (42.4%)	160 (41.7%)	112 (43.4%)	
positive	343 (53.4%)	202 (52.6%)	141 (54.7%)	
missing	27 (4.2%)	22 (5.7%)	5 (1.9%)	
**Menopause**				**0.340**
pre-menopausal	242 (37.7%)	139 (36.2%)	103 (39.9%)	
post-menopausal	400 (62.3%)	245 (63.8%)	155 (60.1%)	
**Age (years)**				**0.061**
< 50	231 (36.0%)	127 (33.1%)	104 (40.3%)	
≥ 50	411 (64.0%)	257 (66.9%)	154 (59.7%)	
**Chemotherapy**				**0.922**
FEC-DG	317 (49.4%)	189 (49.2%)	128 (49.6%)	
FEC-D	325 (50.6%)	195 (50.8%)	130 (50.4%)	

pT, pN, Histology, Grading, ER status, PR status tested using valid categories only (without missing information)

FEC-DG = epirubicin-fluorouracil-cyclophosphamide/gemcitabine-docetaxel chemotherapy

FEC-D = epirubicin-fluorouracil-cyclophosphamide docetaxel chemotherapy

### HER2 phenotype of CTCs and patient characteristics

**Fig 2:**
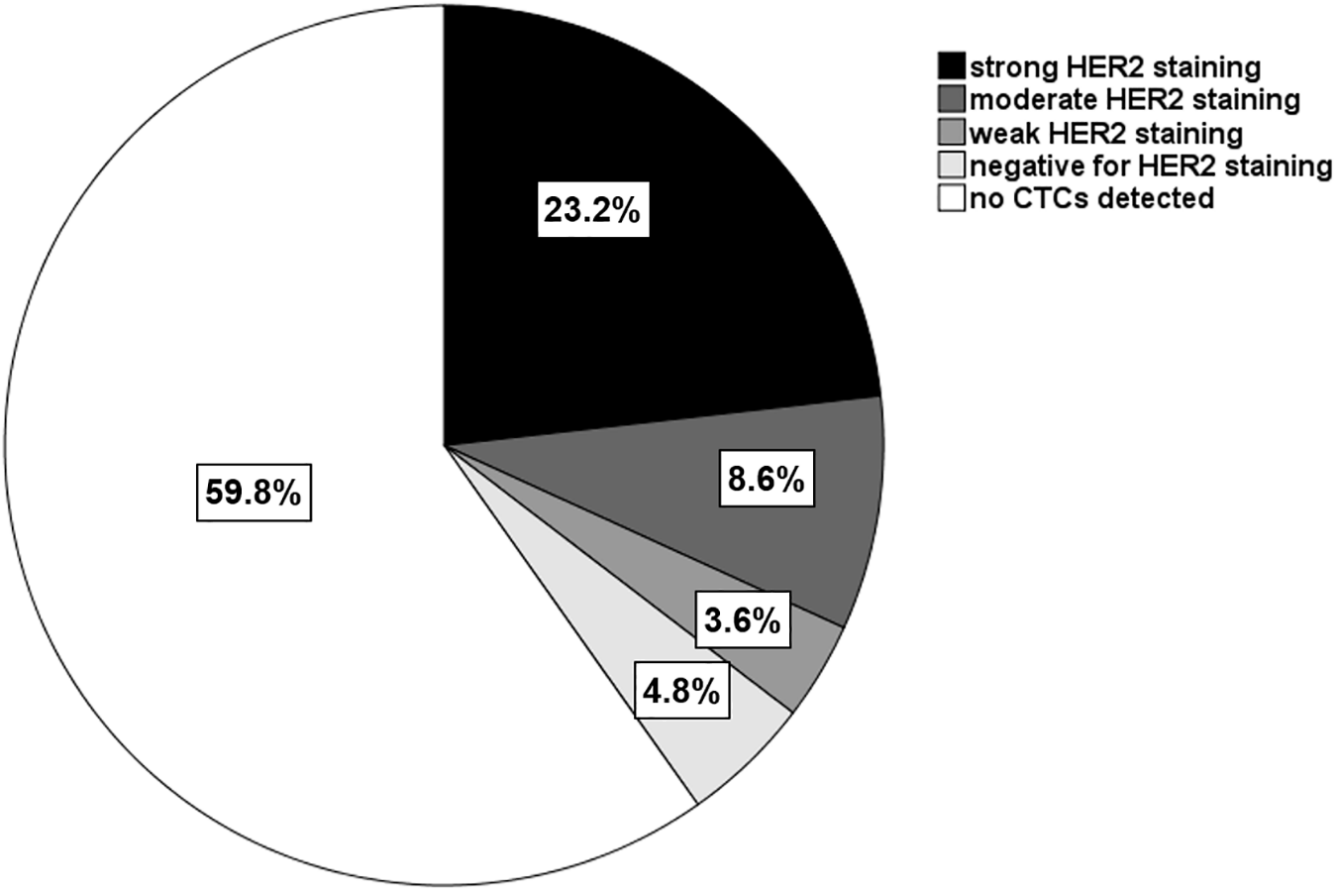
The distribution of samples (n = 642) according to CTC status and the highest HER2 staining intensity of CTCs in the samples.

The HER2 status of CTCs was assessed for all 258 CTC-positive patients. Of these patients, 149 (57.8%) had at least one CTC with strong (+++) HER2 staining; therefore, these patients were classified as having HER2-positive CTCs according to our cutoff criterion. Of the CTC-positive patients, 109 (42.2%) had no CTCs with strong HER2 staining: 55 (21.3%) had at least one CTC with moderate (++) HER2 staining, 23 (8.9%) had no CTCs with moderate (++) HER2 staining but at least one CTC with weak (+) HER2 staining, and 31 (12.0%) were exclusively HER2-negative (-) CTC (Fig 2).

Univariate analyses revealed no association between the presence of HER2-positive CTCs and tumor size, histopathological grading, hormone receptor status or axillary lymph node involvement (chi-squared test, all *p* > 0.05; see Table 2). However, pre-menopausal patients were more likely to have HER2-positive CTCs than post-menopausal patients (chi-squared test, χ^2^ = 4.8, *p* = 0.028), and patients with ductal or lobular invasive breast cancer were less likely to have HER2-positive CTCs than patients with other histological types of BC (chi-squared test, χ^2^ = 6.08, *p* = 0.048). These results were largely confirmed by a multivariate logistic regression analysis with presence of HER2-positive CTCs (yes/no) as binary response variable (see Table 3).

**Table 2:** Associations between presence of HER2-positive CTCs, patient characteristics and clinicopathological characteristics of the primary tumor.

Clinical variable at baseline	CTC-positive (N = 258)		*P*-value
	Only HER2-negative CTCs N = 109	At least one HER2-positive CTC N = 149	
**pT**			**0.335**
1	51 (46.8%)	69 (46.3%)	
2	48 (44.0%)	71 (47.7%)	
3	3 (2.8%)	6 (4.0%)	
4	4 (3.7%)	1 (0.7%)	
missing	3 (2.8%)	2 (1.3%)	
**pN**			**0.430**
0	54 (49.5%)	81 (54.4%)	
+ (1,2,3)	53 (48.6%)	65 (43.6%)	
Missing	2 (1.8%)	3 (2.0%)	
**Histology**			**0.048**
Ductal	50 (45.9%)	54 (36.2%)	
Lobular	7 (6.4%)	4 (2.7%)	
Other	49 (45.0%)	89 (59.7%)	
Missing	3 (2.8%)	2 (1.3%)	
**Grading**			**0.976**
1	1 (0.9%)	1 (0.7%)	
2	40 (36.7%)	54 (36.2%)	
3	66 (60.6%)	90 (60.4%)	
Missing	2 (1.8%)	4 (2.7%)	
**ER status**			**0.626**
negative	39 (35.8%)	58 (38.9%)	
positive	68 (62.4%)	89 (59.7%)	
missing	2 (1.8%)	2 (1.3%)	
**PR status**			**0.263**
negative	43 (39.4%)	69 (46.3%)	
positive	64 (58.7%)	77 (51.7%)	
missing	2 (1.8%)	3 (2.0%)	
**Menopause**			**0.028**
pre-menopausal	35 (32.1%)	68 (45.6%)	
post-menopausal	74 (67.9%)	81 (54.4%)	
**Age (years)**			**0.205**
< 50	39 (35.8%)	65 (43.6%)	
≥ 50	70 (64.2%)	84 (56.4%)	
**Chemotherapy**			**0.201**
FEC-DG	49 (45.0%)	79 (53.0%)	
FEC-D	60 (55.0%)	70 (47.0%)	

pT, pN, Histology, Grading, ER status, PR status tested using valid categories only (without missing information)

FEC-DG = epirubicin-fluorouracil-cyclophosphamide/gemcitabine-docetaxel chemotherapy

FEC-D = epirubicin-fluorouracil-cyclophosphamide docetaxel chemotherapy

**Table 3:** Results of a multivariate logistic regression model with presence of HER-positive CTCs (yes/no) as binary response variable (n = 248 patients; 10 patients were excluded from the multivariate analysis because of missing values).

Clinical variable at baseline	Odds ratio	95% Confidence interval	*P*-value
**pT**			**0.370**
pT2 vs. pT1	0.15	0.67–1.98	0.612
pT3 vs. pT1	2.26	0.44–11.58	0.328
pT4 vs. pT1	0.21	0.02–2.03	0.177
**pN**			**0.541**
pN+ vs pN0	0.85	0.49–1.45	0.541
**Histology**			**0.036**
lobular vs. ductal	0.41	0.10–1.75	0.229
other vs. ductal	1.73	1.00–3.01	0.052
**Grading**			**0.913**
G2 vs. G1	0.73	0.04–14.24	0.835
G3 vs. G1	0.66	0.03–12.92	0.782
**ER status**			**0.710**
positive vs. negative	1.16	0.53–2.52	0.710
**PR status**			**0.337**
positive vs. negative	0.69	0.32–1.48	0.337
**Menopause**			**0.087**
post- vs. pre- menopausal	0.47	0.20–1.12	0.087
**Age (years)**			**0.634**
≥ 50 vs. < 50	1.23	0.52–2.92	0.634
**Chemotherapy**			**0.166**
FEC-D vs. FEC-DG	0.686	0.40–1.17	0.166

FEC-DG = epirubicin-fluorouracil-cyclophosphamide/gemcitabine-docetaxel chemotherapy

FEC-D = epirubicin-fluorouracil-cyclophosphamide docetaxel (D) chemotherapy

### Prevalence of HER2-positive CTCs in relation to the CTC count

**Fig 3:**
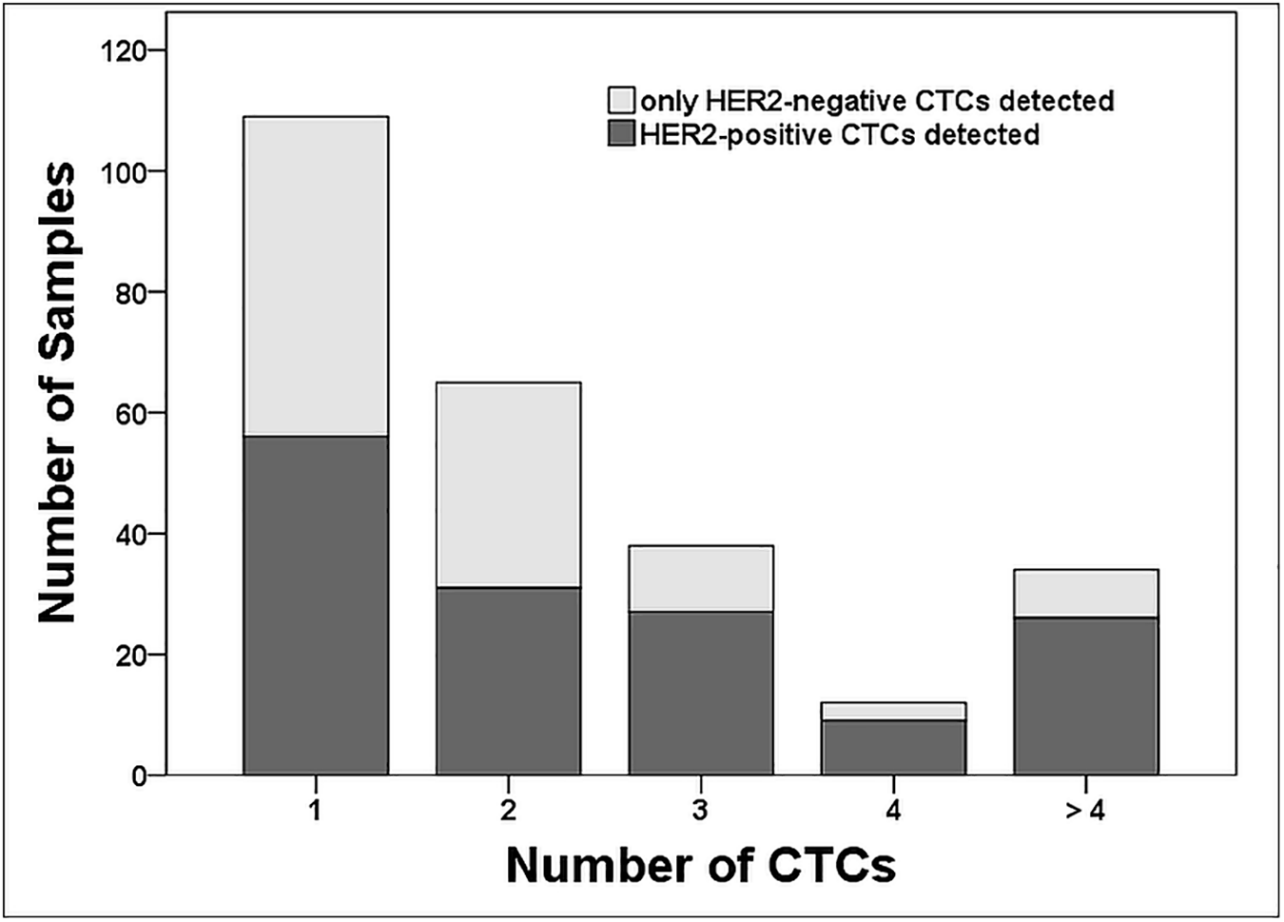
The prevalence of HER2-positive CTCs (i.e., CTCs with strong HER2 staining) in relation to the number of CTCs detected in the blood samples (n = 258).

The probability of detecting at least one HER2-positive CTC increased with the number of detected CTCs (1, 2, 3, 4, or more than 4 CTCs detected; Cochran-Armitage test for trend, *p* = 0.0013; see Fig 3). However, no significant correlation was detected between the number of CTCs in a sample and the percentage of HER2-positive CTCs among these CTCs (Spearman’s rank correlation coefficient r_s_ = -0.07, *p* = 0.264).

### Between-sample and within-sample variability in the HER2 status of CTCs

**Fig 4:**
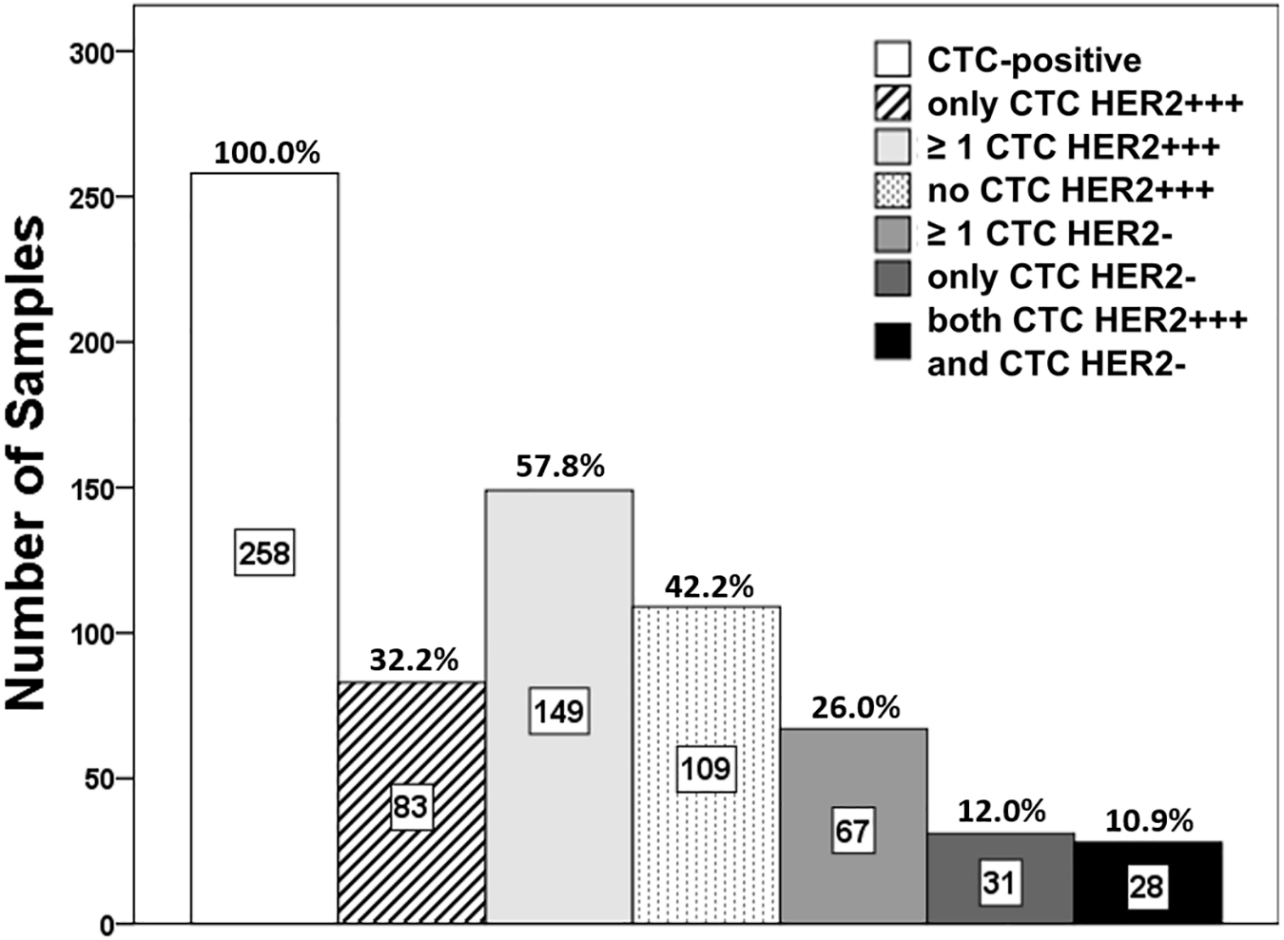
The frequency and percentage (out of 258 CTC-positive samples) of samples with selected characteristics regarding the HER2 status of CTCs.

Detailed data on the between-sample and within-sample variation in the HER2 staining intensities of CTCs are presented in Table 4 and Fig 4. Overall, 56 out of 109 patients (51%) with only one detected CTC had an HER2-positive CTC (i.e., strong (+++) HER2 staining), and 53 patients (49%) had no HER2-positive CTC status (i.e. moderate, weak, or negative HER2 staining). Hence, patients with only one CTC were not more likely to have an HER2-positive CTC than a not HER2-positive CTC (Binomial test, *p* = 0.85). Furthermore, 27 of the 149 patients with more than one CTC exclusively had HER2-positive CTCs; therefore, 83 (32.2%) out of 258 patients with CTCs exclusively had HER2-positive CTCs.

**Table 4:** Between-sample and within-sample variability in the HER2 status of CTCs in patients with early HER2-positive breast cancer.

		Profile of the HER2 status of CTCs (the number of different HER2 staining intensities in the CTCs of a patient)														
		One HER2 staining intensity				Two HER2 staining intensities						Three HER2 staining intensities				Four HER2 staining intensities
**HER2 status of CTCs (HER2 staining intensity)**	**- (negative)**	**+**				**+**	**+**	**+**				**+**	**+**	**+**		**+**
	**+ (weak)**		**+**			**+**			**+**	**+**		**+**	**+**		**+**	**+**
	**++ (moderate)**			**+**			**+**		**+**		**+**	**+**		**+**	**+**	**+**
	**+++ (strong)**				**+**			**+**		**+**	**+**		**+**	**+**	**+**	**+**
**Number of CTCs detected**	**1 (n = 109)**	**19**	**15**	**19**	**56**											
	**2 (n = 65)**	**4**	**6**	**14**	**13**	**2**	**2**	**6**	**6**	**2**	**10**					
	**3 (n = 38)**	**4**		**3**	**10**		**1**	**3**	**3**	**1**	**9**		**1**	**1**	**2**	
	**4 (n = 12)**			**1**	**2**		**1**		**1**		**3**		**2**	**1**		**1**
	**5 or more (n = 34)**	**4**			**2**			**2**	**2**	**1**	**7**	**2**	**2**	**6**	**3**	**3**
	**Total (n = 258)**	**31**	**21**	**37**	**83**	**2**	**4**	**11**	**12**	**4**	**29**	**2**	**5**	**8**	**5**	**4**

Of these 258 patients, 67 (26.0%) had at least one CTC with negative HER2 staining and 31 (12.0%) patients exclusively had HER2-negative CTCs. The majority of these patients had only 1 CTC (n = 19); however, 8 patients had 2–3 CTCs and the remaining patients (n = 4) had 6, 110, 120, and 1,689 CTCs (see Table 4).

Of the 149 patients with more than one CTC, 63 (42.3%) had CTCs with similar HER2 staining intensities and no variation was detected in the HER2 status of the CTCs in these patients. Therefore, within-sample variation in the HER2 staining intensities of CTCs was observed in 86 patients: 62 patients had CTCs with two different HER2 staining intensities, 20 patients had CTCs with three different HER2 staining intensities, and 4 patients had CTCs with four different HER2 staining intensities. Among the 149 patients with more than one CTC, 28 (18.8%) had at least one CTC with negative HER2 staining and at least one CTC with strong HER2 staining (see Table 4 and Fig 4).

## Discussion

We investigated the HER2 expression of CTCs in the peripheral blood of patients with HER2-positive early BC before the start of systemic treatment but after the complete surgical resection of the PT. We did not detect any HER2-positive (+++) CTCs in 42.2% of the CTC-positive blood samples. Overall, 12.0% of patients had HER2-negative (-) CTCs. In addition, 26% of patients had at least 1 HER2-negative (-) CTC, and only 32.2% of patients exclusively had HER2-positive (+++) CTCs. Within-sample variation in the HER2 staining intensities of CTCs was observed in 86 (58%) out of 149 patients with more than one CTC. In several samples, the differences in the HER2 staining intensities were remarkable: 28 (18.8%) out of 149 patients with more than one CTC had at least one CTC with negative HER2 staining and at least one CTC with strong HER2 staining. Furthermore, pre-menopausal patients were more likely to have HER2-positive (+++) CTCs than post-menopausal patients, and patients with ductal or other types of invasive BC were more likely to have HER2-positive (+++) CTCs than patients with the lobular type of BC. Therefore, considerable discordance was found between the HER2 status of the primary tumor and CTCs in early BC. To the best of our knowledge, this study is the largest and most comprehensive to date on the HER2 status of CTCs before adjuvant chemotherapy.

Different approaches are available to enrich and detect CTCs and their phenotype. In this study, we used the CellSearch^®^ System, which is based on immunomagnetic enrichment using EpCAM antibodies and immunofluorescence detection with different antibodies. This system is the most widely used device for CTC detection and was approved by the FDA in 2004 for CTC counting in the management of MBC treatment. The system is associated with high recovery rates and precision even at low cell levels[22]. However, there is currently no standardized and widely accepted method to determine the HER2 status of CTCs. We performed HER2 determination of CTCs using the CellSearch^®^ System according to predefined criteria by Riethdorf et al., which were based on extensive validation experiments that used breast cancer cell lines with a known HER2 gene amplification status (as previously described)[21].

We detected CTCs in 258 (40.2%) patients with early BC before the start of adjuvant chemotherapy. This detection rate is higher than that in our previous findings (21.5% in the adjuvant setting)[18]. However, the patient sample in our previous study comprised patients with HER2-positive and HER2-negative BC, whereas this study only included patients with HER2-positive BC. Therefore, HER2 overexpression by the primary tumor may trigger the prevalence of CTCs, which may be a reason for this discrepancy[23,24].

Using the CellSearch^®^ System, Fehm et al. reported that 42% of patients with HER2-positive MBC exclusively had HER2-negative CTCs and 32% of patients with HER2-negative MBC had HER2-positive CTCs in their peripheral blood[25]. Riethdorf et al. detected HER2-negative and weakly HER2-positive CTCs in 11 of 21 patients with HER2-positive BC before and after the start of neoadjuvant chemotherapy[21]. The data from our study indicate that many CTCs that were detected in patients with HER2-positive early BC before the start of adjuvant chemotherapy did not express HER2. Samples with 5 CTCs or more may not have had any HER2-positive (+++) CTCs or may have exclusively contained HER2-negative (-) CTCs.

Different methods to determine the HER2 status of CTCs have produced comparable results. Using simultaneous immunofluorescence staining of CK, ER and HER2 after the preparation of glass slides, Rack et al. demonstrated that CTCs may have a different hormone and HER2 status than the primary tumor[26]. Georgoulias et al. detected CK19 mRNA-positive CTCs using RT-PCR. Additional immunofluorescence double-staining for CK and HER2 revealed HER2-expressing CTCs in 89% of patients with HER2-negative early BC[27]. Using the AdnaTest, which is based on immunomagnetic cell selection, followed by multiplex PCR, Fehm et al. found that the concordance rates between the ER, PR and HER2 status of CTCs and that of the primary tumor in early BC were 29%, 25% and 53%, respectively[28]. Krishnamurthy et al. evaluated a microfluidic platform for detecting HER2 gene amplification using FISH and CTCs from patients with early BC. These authors detected discordance between the HER2 status of the primary tumor and that of the CTCs in 15% of the patients[29]. In this study, 42% (109 out of 258 CTC-positive patients) of the patients with HER2-positive early BC had no HER2-positive (+++) CTCs. Therefore, irrespective of the method used, a high discordance rate in the HER2 expression between the primary tumor and CTCs was found in all of these studies.

Intratumoral heterogeneity in HER2 expression may explain unexpected failures of HER2-targeted therapy with trastuzumab[30]. Ohlschlegel et al. demonstrated that HER2 heterogeneity frequently occurred in breast cancer according to the ASCO/CAP definition and occurred most often in carcinomas with equivocal (2+) IHC results for HER2[7]. By studying different areas of one tumor, the discordance rates of HER2 amplification among the analyzed samples ranged from 36% to 54%[6]^,^[31]. Primary tumors with HER2 heterogeneity may shed CTCs with different HER2 staining levels which may explain our findings. The primary tumors of our patient collective were tested for HER2 amplification according the ASCO/CAP criteria and fulfilled the criteria for HER2-targeted therapy. In clinical routine though the primary tumor is not tested for HER2 heterogeneity. Therefore CTCs may give an even more precise picture of the actual HER2 status of one patient.

Few studies have demonstrated the effects of HER2-targeted therapies on the number of detected CTCs[27,32]. However, no data are available on the effect of HER2-targeted therapies on the HER2 status of CTCs. This issue will be addressed in ongoing trials that are focusing on personalized treatment that is based on the HER2 phenotype of CTCs and the effect of targeted therapies on the number and phenotypes of CTCs. The DETECT III trial is currently investigating an HER2-targeted therapy (lapatinib) in patients with HER2-negative MBC but HER2-positive CTCs. In the adjuvant setting, the Treat CTC trial will evaluate the effect of an HER2-targeted therapy with trastuzumab on CTC load in patients with persistent CTCs after adjuvant chemotherapy,[33] irrespective of the HER2 status of CTCs.

Our study focused on a highly relevant topic of research; however, there are several limitations to this study. Detecting the HER2 status of CTCs remains the focus of ongoing investigation. There are currently no fully automated methods for determining the HER2 status of CTCs, and the final evaluation of HER2 status in this study was based on the subjective interpretation of the investigator. Furthermore, a high degree of discordance (26%) was found in the HER2 testing of the solid tumor tissue between local and central laboratories. The need to use high-volume, experienced laboratories for HER2 testing is supported by the high degree of agreement (89.1%) between central and reference laboratories[34]. Therefore, our findings may have been affected by the lack of reliability in the HER2 testing of the primary tumor.

Additionally, the CTC detection rate was low (20%-40%)[18,21,35] in the neo-/adjuvant setting compared with that in the metastatic setting (50%-80%)[15,17,36]. In several studies of patients with early BC, only one CTC could be detected in the majority of the blood samples[18,21,37], which makes the evaluation of the HER2 status of CTCs even more challenging. Therefore, we pooled three CellSave tubes to reach a total volume of 30ml to increase the detection rate and the sensitivity.

Furthermore, these are data describing the prevalence of CTCs only. Prognostic influence of CTCs in early HER2-positive BC will be presented as soon as the follow up within the SUCCESS B trial will be completed.

## Conclusion

In conclusion, this study is the largest to date that has evaluated the HER2 status of CTCs before adjuvant chemotherapy. This study found that the HER2 status of CTCs may differ from the HER2 status of the primary tumor in patients with early HER2-positive BC. Our results strongly suggest that the HER2 status of patients needs to be reassessed to correctly determine the appropriate use of HER2-targeted therapy. Ongoing trials are evaluating the phenotype and molecular characterization of CTCs as a basis for decision-making in targeted drug development and individualized treatment.

## Supporting information

S1 File Modified ficoll Pre-processing procedure for 30 mL of whole blood prior to CellSearch™ circulating tumor cell test. (PDF)

S2 File SUCCESSB_DATA_PLOS_ONE.xlsx. (XLSX)
