# Supplementary material for: The HER2 phenotype of circulating tumor cells in HER2-positive early breast cancer: A translational research project of a prospective randomized phase III trial
Source: PLoS One. 2017 Jun 6;12(6):e0173593. doi: 10.1371/journal.pone.0173593 (PMC5460789; doi:10.1371/journal.pone.0173593)
Supplement: S1 File — (PDF) [file pone.0173593.s001.pdf]

Poster Session
5th International Symposium on Minimal Residual Cancer
San Francisco, California September 2005

Modified Ficoll Pre-processing Procedure for 30 mL of Whole Blood Prior to CellSearch™ Circulating Tumor Cell Test

Steve Gross, Thai Bui, Chandra Rao, Mark Connelly, and Leon W.M.M. Terstappen
Immunicon Corporation, Huntingdon Valley, PA

Abstract

Purpose Statement: The presence of 5 or more circulating tumor cells (CTCs) in 7.5 mL of blood detected by the CellSearch™ System predicts shorter progression-free survival (PFS) and overall survival (OS) in patients with metastatic carcinoma. The number of CTCs is often low, even in metastatic disease. In patients with early stage disease, the frequency of detection and the number of CTCs is low. We developed a modified ficoll method to pre-process a larger starting volume (30 mL) of blood prior to introducing the sample to the CellTracks® AutoPrep System for CTC enrichment. This method was designed to isolate more CTCs in order to facilitate characterization of these cells and to elucidate their role in the cancer disease process. The objective of this study was to determine the increase in sensitivity and yield of CTCs using this new method.

Brief Description: The standard CellSearch™ procedure was used as the baseline for comparing a standard ficoll enrichment procedure and modified ficoll method, both using 30 mL of blood. Summary of the CellSearch™ procedure: 7.5 mL of blood is processed with the CellSearch™ CTC Kit using the CellTracks AutoPrep System and the enriched sample is counted using the CellTracks Analyzer II. Comparative methods: 30 mL samples were pre-processed using a standard ficoll procedure using Histopaque 1.083 or by the modified ficoll procedure. If recovery were perfect, the 30 mL samples would yield 4-fold recovery compared to the 7.5 mL sample processed with the CellSearch™ System. Seven tubes (approximately 70 mL) of blood was drawn into CellSave® Preservative Tubes and pooled. Cells from the cell lines SKBR, PC3-9, MCF-7, and COLO-205 were spiked into the pooled blood and separated into 30 mL and 7.5 mL fractions. Cell recovery was compared to the 7.5mL CellSearch™ samples. Blood from metastatic cancer patients were used to compare CTC frequency between methods.

Summary: No CTCs were detected in blood from 20 healthy donors with any of the methods. Recovery compared to the CTCs isolated by the CellSearch™ system from 7.5 mL were as follows: The standard 30 mL ficoll method yielded 2.7-fold with SKBR-3 cells, 2.6-fold with PC3-9 cells, 2.9-fold with MCF-7cells, and 2.9-fold with COLO-205 cells. The modified ficoll procedure gave recoveries of 3.6-fold with SKBR-3 cells, 3.4-fold with PC3-9 cells, 3.2-fold with MCF-7cells, and 3.2 fold with COLO-205 cells. Regression analysis of SKBR-3 cells spiked at 5, 10, 50, and 100 into 30mL blood from 7 donors using the modified ficoll procedure generated a slope = 0.786 with an R^2 = 0.986. The modified ficoll procedure was used to pre-process samples from 63 patient samples. 14 (22.2%) had $\geq$ 1 CTC in 7.5 mL blood (mean 82.9 median 1.5), and 22 (35.0%) had $\geq$ 1 CTC in 30 mL blood (mean 148.4 median 2.0) and yielded a mean of 3.6-fold recovery $\pm$1.3 (median 3.1). 9 (14%) patients with 0 CTCs in 7.5mL had $\geq$ 1CTC in 30mL and 2 (3%) patients with 0 CTCs in 30mL had 1 CTC detected in 7.5mL.

Conclusion: A modified ficoll procedure reduces 30 mL of blood to a 7.5 mL volume that can be processed on the CellTracks® AutoPrep System. In blood from metastatic carcinoma patients, the yield of CTCs increased 3.6-fold and the sensitivity increased from 22 to 35%.

Methods

Specimen Collection and Enumeration of CECs

Sample Collection and Preservation

- Collect blood into CellSave® Preservative Tubes (Immunicon, Huntingdon Valley, PA).

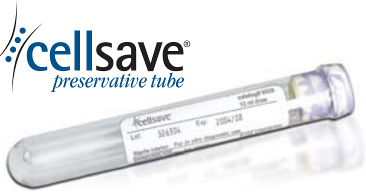

Reagent Kits

- CellSearch™ Circulating Tumor Cell Kit (Veridex, LLC) Transfer 7.5 mL blood to 15 mL CellTracks® AutoPrep sample tubes that are provided with the CellSearch Kit. This kit contains reagents and supplies for 16 tests including:
  - Anti-EpCAM Ferrofluid: to capture epithelial cells
  - Nucleic Acid Dye: DAPI to identify nucleated cells
  - Capture Enhancement Reagent: to maximize capture efficiency irrespective of variable expression of EpCAM antigen
  - Permeabilization Reagent: to allow for staining of intracellular antigens.
  - Cell Fixative: to fix cells in the final samples.
  - Staining Reagent: anti-CK-PE/CD45APC.

CellSearch™
Circulating Tumor Cell Kit

- Place reagent kit and up to 8 samples on the CellTracks® AutoPrep System.
- Follow the instrument prompts to set up a batch.

celltracks® autoprep system

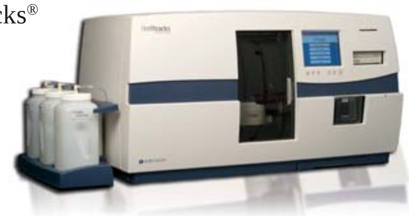

Standardized, Automated Sample Processing

Analysis

- Analyze the samples using the CellTracks® Analyzer II. This semi-automated fluorescent microscope captures images with a 10x objective for each of the four fluorescent filter cubes, covering the entire surface of the cartridge. The gallery is presented based on the test definition, which is determined by the kit and Marker reagent used.

celltracks® analyzer II

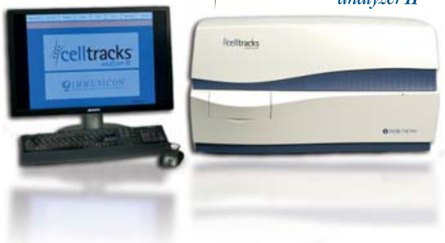

Phenotype a Circulating Tumor Cell EpCAM+/DAPI+/CK-PE+/CD45APC-

Check Composite image to count the event as a CTC

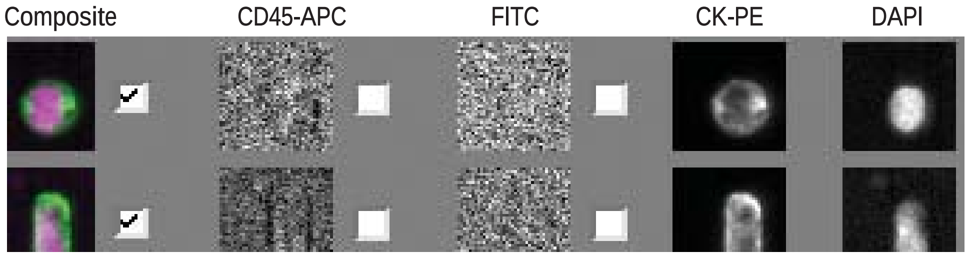

Overview of Pre-Processing Procedures

Standard Ficoll Procedure

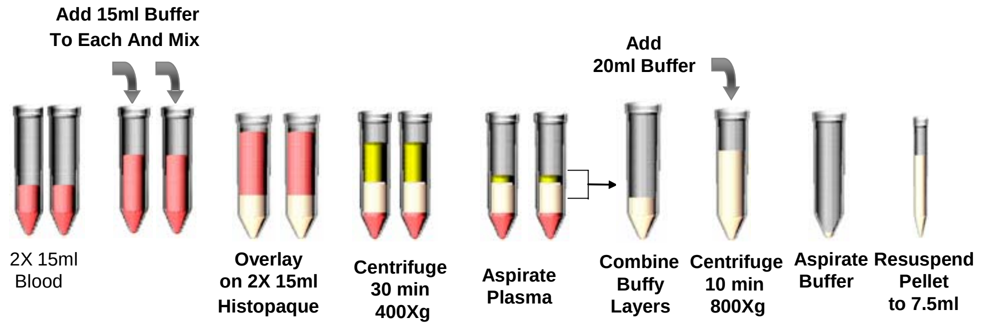

Modified Ficoll Procedure

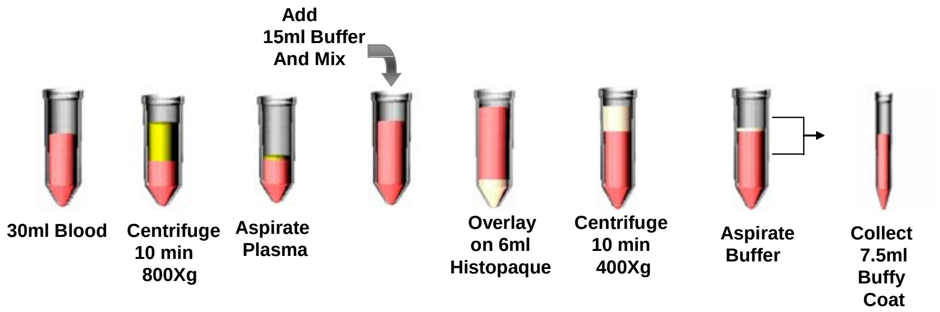

1. Centrifuge 30 mL of blood collected into CellSave® Tubes. Aspirate plasma.
2. Add Dilution Buffer. Layer diluted blood over Histopaque-1.083
3. Centrifuge. Aspirate buffer.
4. Aspirate 7.5 – 8.0 mLs from the top of the sample (includes the buffy coat). Dispense into to a CellTracks® AutoPrep sample tube.
5. Process samples using the CellTracks® AutoPrep System and the CellSearch™ CTC Kit.

Materials Needed

Equipment:

- 5 mL standard serlogical pipet, (Fisher cat# 13-678-12D or equivalent)
- Drummond Pipet-Aid (or equivalent)
- (For cell culture model systems) 5% CO_2 cell culture incubator, CEDCO, Model 1400)
- Centrifuge, Beckman model, #1190, (or equivalent)
- 50 mL centrifuge tubes, (Fisher cat# 05-539-7)
- CellTracks® AutoPrep sample tube (from CellSearch™ CTC Kit, Veridex cat. #7900001)
- Variable flow mini-pump (Fisher cat# 13-876-1 or equivalent)
- 9" disposable Pasteur pipets, (Fisher cat# 13-678-200 or equivalent)
- Rubber bulbs, (Fisher cat# 14-065 B, or equivalent)
- 30ml syringe, (Becton Dickinson cat# 309650)
- Valve and Tubing

Reagents and Buffers:

- Dilution Buffer (Immunicon cat# 7016)
- Histopaque-1.083, (Sigma cat# 1083-1)

Test Procedure

1. Collect >30 mL blood aseptically into four (4) CellSave® Preservative Tubes only.
2. Pool CellSave® Tubes into a 50 mL centrifuge tube.
3. Transfer 30 mL of blood into a fresh 50ml centrifuge tube.
4. Centrifuge for 10 minutes at 800xg. **Make sure centrifuge brake is off.**
5. Aspirate plasma down to within 2-2.5 mL of the buffy coat. Be careful not to disturb buffy coat.

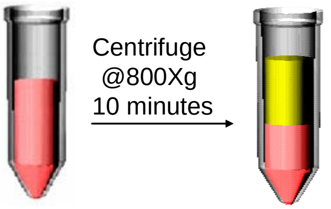

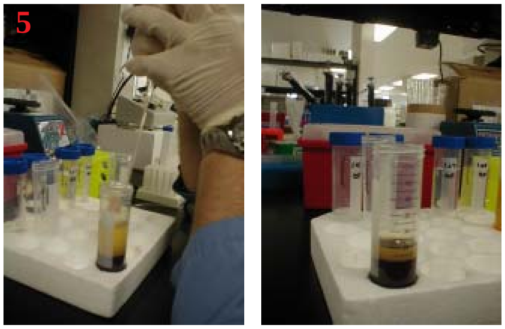

6. Add 15 mL Dilution Buffer and mix by gentle inversion eight (8) times.
7. Add 6 mL Histopaque-1.083 to a fresh 50 mL centrifuge tube.
8. Assemble 30 mL syringe with valve and tubing. Place the end of the tubing against the inner wall of the 50 mL centrifuge tube containing 6 mL Histopaque prepared in step 7. Pour blood mixture from step 6 into 30ml syringe. **Make sure valve is closed.**
9. Open valve until blood mixture begins to **slowly** flow. Blood should flow down the inner wall of centrifuge tube to avoid any mixing of blood and Histopaque. Layer the entire blood mixture onto the Histopaque then close valve.

The steps involving layering the diluted sample over Histopaque may be performed manually, but recovery is maximized by carefully controlling the flow of blood over the Histopaque.

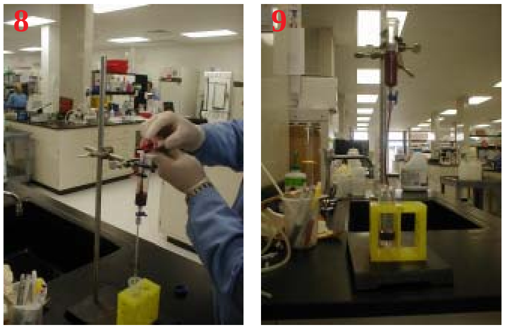

10. Centrifuge for 10 minutes at 400Xg. **Make sure centrifuge brake is off**.
11. Manually aspirate buffer down to within 2-2.5mls of the buffy coat taking care not to disturb buffy coat.
12. Mark the the CellTracks® AutoPrep sample tube at 7.5 mL. Remove the cotton from a 5 mL pipette and assemble the tip to tubing and to a variable flow mini-pump. Place the upside down pipette at the surface of the sample, in the center of the tube. Collect 7.5 mL of sample from the top to the tube, which includes the buffy coat.
13. Transfer to a CellTracks® AutoPrep sample tube and process on the CellTracks® AutoPrep System. Run the sample using the CTC Control protocol.

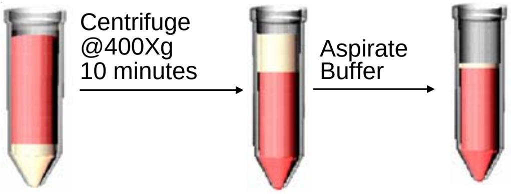

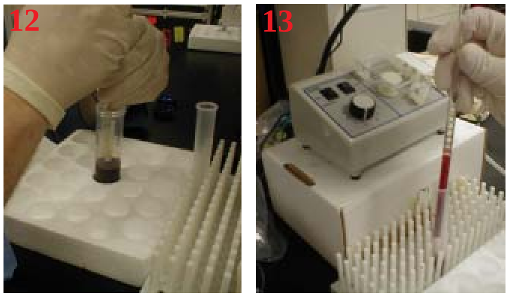

Aspirating the buffy coat in a controlled fashion is critical to recovery. Set the peristaltic pump to a low setting (5 mL/min). Aspirate from the center of the 50 mL tube. Dispense into the CellTracks® AutoPrep sample tube by reversing direction.

Mean Fold Recovery of Cells From 30mls Blood vs. 7.5mls Blood

Three methods were compared to the standard 7.5 mL CellSearch procedure in spiking experiments. The flotation and modified ficoll methods were compared using patient samples.

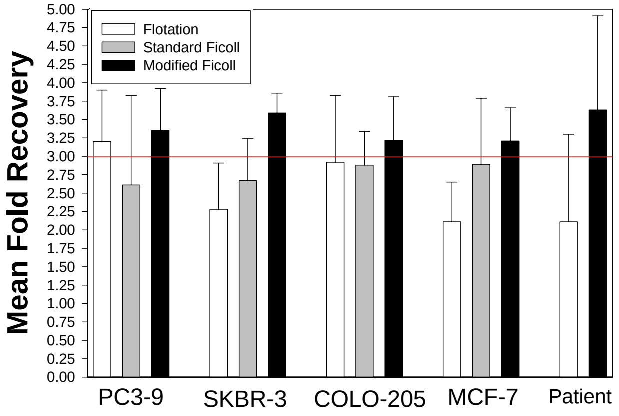

Linearity using Modified Ficoll.

Recovery of 5, 10, 50, & 100 SKBr-3 Cells Spiked into 30mls Blood from 7 Donors

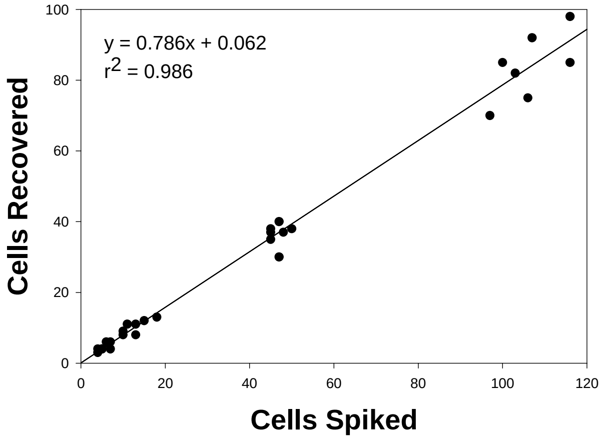

CTCs Detected from Patient Samples

The modified ficoll method was used to analyze recovery of tumor cells in patient samples.

Sample	Carcinoma	CTC/7.5ml Blood	CTC/30ml Blood
1	Colon	**0**	**2**
2	Lung	**0**	**1**
3	Colon	**1**	**6**
4	Breast	**0**	**1**
5	Parotid	**0**	**1**
6	Ovarian	**1**	**1**
7	Breast	**0**	**10**
8	Breast	**1**	**5**
9	Colon	**0**	**1**
10	Colon	**1**	**0**
11	Parotid	**1**	**0**
12	Skin	**1**	**2**
13	Prostate	**0**	**1**
14	Unknown	**5**	**12**
15	Colon	**4**	**17**
16	Breast	**0**	**3**
17	Breast	**0**	**1**
18	Stomach	**1081**	**3010**
19	Colon	**2**	**6**
20	Breast	**0**	**2**
21	Breast	**51**	**160**
22	Lung	**5**	**16**
23	Breast	**1**	**1**
24	Colon	**5**	**18**

Normal Subjects

	N	≥1 CTC
7.5ml Blood	20	0
30ml Blood	20	0

Cancer Patients

	N	≥1 CTC	% of Total	≥5 CTC	% of Total	≥10 CTC	% of Total	≥100 CTC	% of Total	Mean CTC	Median CTC
7.5ml Blood	63	14	22	5	8	2	3	1	1.5	82.9	1.5
30ml Blood	63	22	35	10	16	7	11	2	3	148.4	2

Samples with no CTCs detected in both 7.5 and 30mL are not shown in the detail table of patient samples.

Conclusions

- 30 mL of blood can be processed by a modified ficoll procedure that can be further enriched for CTCs on the CellTracks® AutoPrep System.
- Increasing blood volume from 7.5 mL to 30 mL does not increase background.
- A four-fold increase in blood volume from metastatic cancer patients increased the yield of CTCs by 3.6 fold.
- Increasing the volume of blood will allow for greater yields of rare circulating epithelial cells in cancer patient samples.

IMMUNICON®
3401 Masons Mill Road, Suite 100
Huntingdon Valley, Pennsylvania 19006
phone 215.830.0777, 877.822.0777
fax 215.830.0751
web www.immunicon.com
